# The effect of the COVID-19 pandemic on the epidemiology of positive blood cultures in Swiss intensive care units: a nationwide surveillance study

**DOI:** 10.1186/s13054-021-03814-z

**Published:** 2021-11-22

**Authors:** Lauro Damonti, Andreas Kronenberg, Jonas Marschall, Philipp Jent, Rami Sommerstein, Marlieke E. A. De Kraker, Stephan Harbarth, Michael Gasser, Niccolò Buetti

**Affiliations:** 1 Department of Infectious Diseases, Inselspital, Bern University Hospital, University of Bern, Bern, Switzerland; 2 Division of Infectious Diseases, Regional Hospital Lugano, Lugano, Switzerland; 3 Swiss Centre for Antibiotic Resistance, Institute for Infectious Diseases, University Bern, Bern, Switzerland; 4 Department of Health Science and Medicine, University of Lucerne, Lucerne, Switzerland; 5 Infection Control Program and WHO Collaborating Centre on Patient Safety, Geneva University Hospitals and Faculty of Medicine, Geneva, Switzerland; 6 Infection Control Programme, University of Geneva Hospitals and Faculty of Medicine, WHO Collaborating Center, Geneva, Switzerland; 7 University of Paris, INSERM, IAME, Team DeSCID, Paris, France

**Keywords:** COVID-19, ICU, Bloodstream infections, Blood culture contaminations, Surveillance

## Abstract

**Background:**

Evidence about the impact of the pandemic of COVID-19 on the incidence rates of blood cultures contaminations and bloodstream infections in intensive care units (ICUs) remains scant. The objective of this study was to investigate the nationwide epidemiology of positive blood cultures drawn in ICUs during the first two pandemic waves of COVID-19 in Switzerland.

**Methods:**

We analyzed data on positive blood cultures among ICU patients, prospectively collected through a nationwide surveillance system (ANRESIS), from March 30, 2020, to May 31, 2021, a 14-month timeframe that included a first wave of COVID-19, which affected the French and Italian-speaking regions, an interim period (summer 2020) and a second wave that affected the entire country. We used the number of ICU patient-days provided by the Swiss Federal Office of Public Health as denominator to calculate incidence rates of blood culture contaminations and bloodstream infections (ICU-BSI). Incidence rate ratios comparing the interim period with the second wave were determined by segmented Poisson regression models.

**Results:**

A total of 1099 blood culture contaminations and 1616 ICU-BSIs were identified in 52 ICUs during the study. Overall, more episodes of blood culture contaminations and ICU-BSI were observed during the pandemic waves, compared to the interim period. The proportions of blood culture contaminations and ICU-BSI were positively associated with the ICU occupancy rate, which was higher during the COVID-19 waves. During the more representative second wave (*versus* interim period), we observed an increased incidence of blood culture contaminations (IRR 1.57, 95% CI 1.16–2.12) and ICU-BSI (IRR 1.20, 95% CI 1.03–1.39).

**Conclusions:**

An increase in blood culture contaminations and ICU-BSIs was observed during the second COVID-19 pandemic wave, especially in months when the ICU burden of COVID-19 patients was high.

**Supplementary Information:**

The online version contains supplementary material available at 10.1186/s13054-021-03814-z.

## Background

The pandemic of severe acute respiratory syndrome coronavirus 2 (SARS-CoV-2)-related infection (COVID-19) is affecting health systems worldwide. In Switzerland, a first wave of the COVID-19 pandemic predominantly involved the French and Italian-speaking regions over the period February to May 2020, while a second wave started in October 2020 and affected the entire country until May 2021. During these months, hospitals, and in particular intensive care units (ICUs) had to deal with several challenges, such as patient overflow, staff reinforcements by physicians and nurses with limited or no experience in intensive care practices, and, especially during the first wave, transient or sustained shortages in personal protective or supportive equipment [1, 2]. Whether the overall management of critically ill patients (both with and without COVID-19) during the outbreaks was adequate, remains an open question. An important proxy for adequate ICU care includes the rate of bloodstream infections (BSI), and especially contamination rates of blood cultures. However, evidence remains scant about the impact of the pandemic on the epidemiology of bloodstream infections [3–9], especially in intensive care units (ICU-BSI) [10–12]. The objective of this study was to investigate the nationwide epidemiology of positive blood cultures drawn in ICUs during the first two pandemic waves of COVID-19 in Switzerland with a special focus on blood culture contamination.

## Methods

### Study setting and design

We analyzed national, epidemiological and microbiological data on positive blood cultures among ICU patients prospectively collected by the Swiss Centre for Antibiotic Resistance (ANRESIS) and epidemiological data provided by the COVID-19 surveillance conducted by the Swiss Federal Office of Public Health (FOPH) from March 30, 2020, to May 31, 2021.

### Data sources

ANRESIS regularly receives information on all positive blood cultures from over 30 Swiss microbiology laboratories, some of them collecting data from multiple hospitals. Hospitals are distributed across the country and represent at least 80% of annual hospitalization days [13]. Species identification and antimicrobial susceptibility testing are based on tests performed in the local laboratories, which apply either European Committee on Antimicrobial Susceptibility Testing (EUCAST, https://eucast.org) or Clinical and Laboratory Standards Institute (CLSI, https://clsi.org) guidelines. All laboratories are participating in at least one external quality program of either the Swiss quality control program issued by the Institute for Medical Microbiology, University of Zürich (http://www.imm.uzh.ch/services/qc.html), or the National External Quality Assessment Service (NEQAS; https://ukneqas.org.uk/). We included data on ICU isolates from those Swiss hospitals that sent information on a regular basis during the entire study period. Included hospitals are listed in Additional file 1: Fig. S1. Variables routinely collected by ANRESIS include sex, date of detection of the episode, detected microorganism species, hospital type (non-university *versus* university) and location (city and canton).

Starting March 30, 2020, the FOPH collected detailed data on Swiss hospital’s occupancy regarding patients with and without COVID-19. Aggregated data on the daily number of hospitalized ICU patients (with and without COVID-19) as well as ICU capacity and occupancy (in percent) in each center were recorded by the cantonal health authorities and then transmitted to the FOPH. Data are freely available on the website of the FOPH (https://www.covid19.admin.ch).

### Definitions

A single blood culture episode was defined as one or more positive blood cultures with the same microorganism from the same patient within 14 days. Episodes were recorded in patients hospitalized in the ICU and were considered as either *blood culture contamination* or *ICU-BSI*. Possible skin contaminants were identified according to the Centers for Disease Control and Prevention (CDC) National Healthcare Safety Network (NHSN) Patient Safety Component Manual [14]. Growth of possible contaminants was considered to represent BSI, if the same species was detected at least twice within the first two days of a new episode, otherwise, the episode was accounted as a blood culture contamination. The duration of an episode was fixed to 14 days, whether it was judged as infection or contamination. Non-contaminant episodes were considered as bloodstream infections (BSI), thus including bacterial and fungal isolates [15]. Species responsible for ICU-BSI were grouped in the following nine classes: *Staphylococcus aureus*, *Streptococcus* spp, *Enterococcus* spp. *Escherichia coli*, *Pseudomonas* and other non-fermenters, other Gram-negative bacilli (GNB), anaerobes, *Candida* spp, and other, which included coagulase-negative Staphylococci (CoNS).

### Study periods

The study was split in three periods. The first period (“first wave”) started on March 30, 2020, with the beginning of the FOPH surveillance and ended on May 11, 2020, with the cessation of the extraordinary measures previously imposed by the Swiss Federal Council. The second period (“interim period”), from May 12, 2020, to October 18, 2020, coincided with a period with extremely few cases of COVID-19 related hospitalizations. The third period (“second wave”) started on October 19, 2020, when new extraordinary measures were imposed due to the increased number of cases, and ended on May 31, 2021, the end of the study.

### Outcomes

The primary outcomes of this study were blood culture contamination and BSI among ICU patients during three periods of the of COVID-19 pandemic. Secondary outcomes included the occurrence of the specific microorganisms responsible for the episodes and the description of the patient population.

### Statistical analysis

The statistical analysis included five steps. First, baseline characteristics of the patients related to the episodes depending on pandemic period were compared with chi-square and Kruskal–Wallis tests, as appropriate. Second, we performed descriptive analyses of the whole study period focusing on monthly total number and incidence (*i.e.*, using the number of patient-days as denominator) of blood culture contaminations and ICU-BSI; subsequently, we depicted the correlations of the ICU occupation due to COVID-19 patients with the incidence of blood culture contaminations, incidence of ICU-BSI and with the proportion of blood culture contaminations. For this analysis, data were previously inspected using Q-Q plots and Shapiro–Wilk tests in order to assure that the normality distribution assumption is met. A Pearson's correlation coefficient was then calculated and a Pearson correlation tests was performed. Third, we graphically displayed the microorganisms responsible for the episodes. Fourth, incidence rate ratios [IRR] for blood culture contaminations, ICU-BSI and specific microorganisms between the interim and second wave were evaluated by segmented Poisson regression models using aggregated monthly data, and patient-days as off-set. Since the surveillance of the FOPH on the hospital occupation during the COVID-19 pandemic started on March 30, 2020, data about the first wave were incomplete and were therefore excluded from this analysis. Overdispersion was tested using the likelihood ratio test with subsequent fitting of a negative binomial model, if required. Finally, we performed a confirmatory analysis for blood culture contamination using a Poisson regression model investigating the effect of the second wave and introducing the time and the interaction between time and second wave.

All analyses were performed with R (version 4.0.2) and SAS (version 9.4). *p* values < 0.05 were considered statistically significant. The analysis is in compliance with the STROBE guidelines for observational studies [16].

### Ethics statement

As the analysis has been performed on anonymized non-genetic surveillance data, ethical consent was not required according to the Swiss law for research on humans (Article 33, Paragraph 2, Human Research Act).

## Results

### Patient population and epidemiological description of blood culture contaminations and ICU-BSI

During the study, 29′270 blood culture episodes were reported to ANRESIS, of which 2′912 were collected among ICU patients. A total of 287 episodes were excluded, either because identified among children under 16 years of age (*N* = 237) or from patients abroad (*N* = 50). Of the 2′715 remaining episodes collected in 52 ICUs, 1099 were considered as blood culture contaminations and 1616 as ICU-BSIs, according to the aforementioned definitions (Additional file 1: Fig. S2). Baseline characteristics of the patients are listed in Table 1, stratified according to the period of the study. Of note, positive blood cultures recorded during the second wave (*versus* the first wave) were more frequently detected in older patients hospitalized in university hospitals. At the same time, blood culture contamination proportions (contrary to ICU-BSI proportions) were higher in the two waves than in the interim period.

**Table 1:** Main baseline characteristics of all positive blood cultures included in the study stratified with respect of the period of the pandemic

	First wave March 30**–**May 11 2020	Interim period May 12**–**October 18 2020	Second wave October 19 2020**–**May 31 2021	*p* value*
Total number of episodes	*n* = 301	*n* = 753	*n* = 1661	
Age ≥ 60 years, *n* (%)	147 (48.8)	434 (57.6)	994 (59.8)	< 0.01
Female sex, *n* (%)	83 (27.6)	206 (27.4)	492 (29.6)	0.47
Episodes originating at a university hospital, *n* (%)	74 (24.6)	308 (40.9)	528 (31.8)	< 0.01
Blood culture contaminations *n* (%)	125 (41.5)	268 (35.6)	706 (42.5)	< 0.01

*Proportion comparison between the different groups performed with the Chi-square test

### Graphical description of blood culture contaminations, ICU-BSI and specific microorganisms during the study

**Fig. 1:**
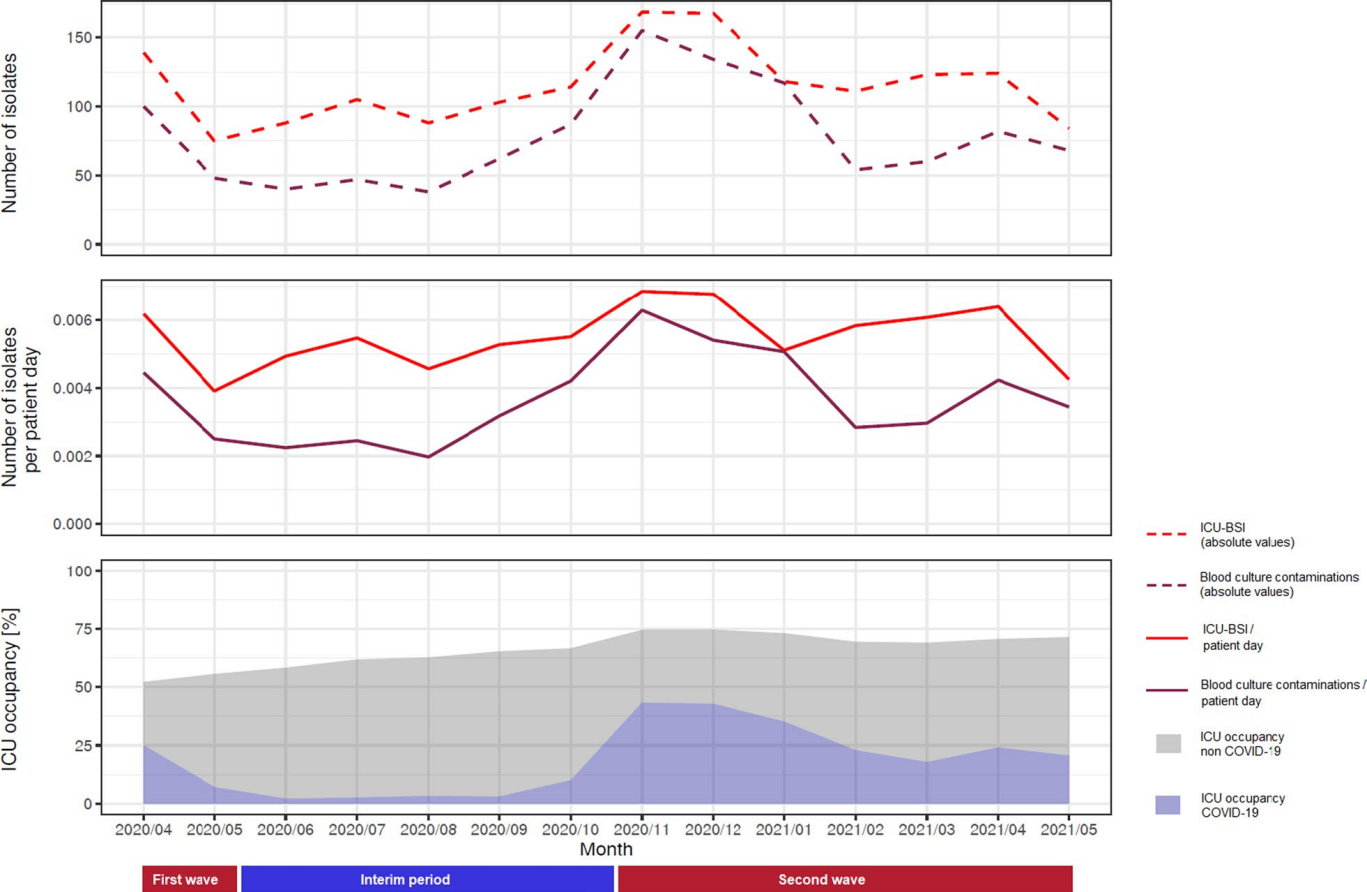
Temporal trends of blood culture contaminations, ICU–BSI, and ICU occupancy. Upper panel: Absolute numbers of ICU–BSI (red dashed line) and blood culture contamination (purple dashed line). Middle panel: Incidence rates of ICU–BSI (red line) and blood culture contamination (purple line). Lower panel: ICU occupancy due to COVID-19 patients (blue) and COVID-19-negative patients (grey)

In Fig. 1, we show the temporal trends of the crude number of ICU–BSI and blood culture contaminations (upper panel), their incidence rates per patient-day (middle panel) and the ICU occupancy due to COVID-19 and COVID-19-negative patients (lower panel). Higher numbers and incidence rates of blood culture contaminations and ICU-BSI were observed during the two waves, and followed a similar pattern as the ICU occupancy on a national level due to COVID-19 patients.

**Fig. 2:**
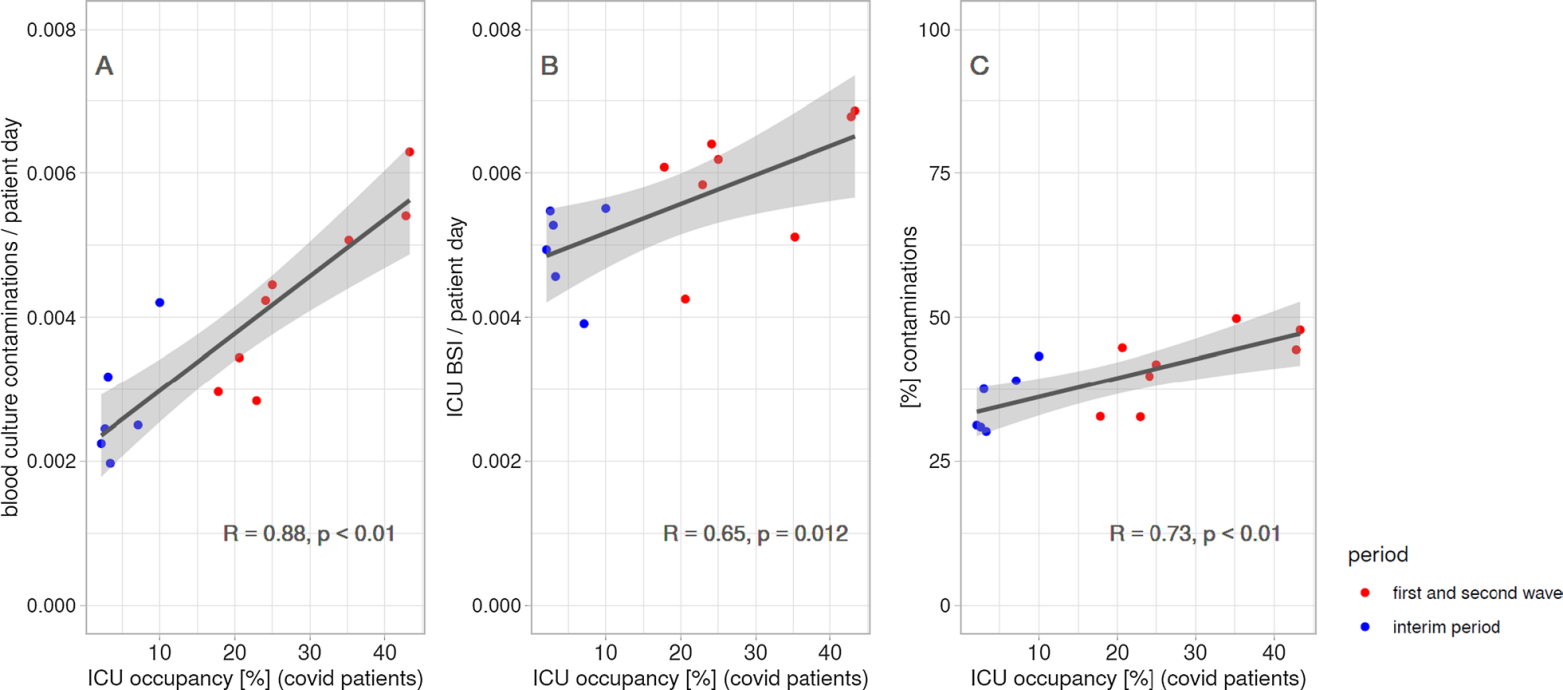
Correlations of the ICU occupation due to COVID-19 patients with blood culture contaminations incidence (**a**), ICU-BSI incidence (**b**) and percentage of blood culture contaminations (**c**). Each dot represents data from one month. In each panel, a Pearson's correlation coefficient ("*R*"), a *p* value of a Pearson correlation test ("*p*"), a univariate regression line and the corresponding 95% confidence interval (gray area) are shown

In Fig. 2, we show the correlations of the ICU occupation due to COVID-19 patients with incidence of blood culture contaminations (Panel A), ICU-BSI incidence (Panel B) and percentage of blood culture contaminations (Panel C). Overall, a significant, positive association was detected between both blood culture contamination and ICU-BSI with ICU occupation, which was higher during the COVID-19 waves. In Additional file 1: Fig. S3, these correlations were evaluated with respects of the ICU occupation due to all patients, showing similar trends with, however, a less significant association when compared to the occupation due to COVID-19 patients only.

Additional file 1: Fig. S4 provides information about the weekly count of the episodes recorded by ANRESIS. For a better understanding, we included in this chart the last two pre-pandemic years and as well as the whole study period. The increase in episodes during the first and the second wave was mainly driven by contaminant microorganisms (in particular coagulase negative staphylococci, CoNS).

### Incidence of blood culture contaminations and ICU-BSI, and specific microorganisms between interim period and second wave

**Fig. 3:**
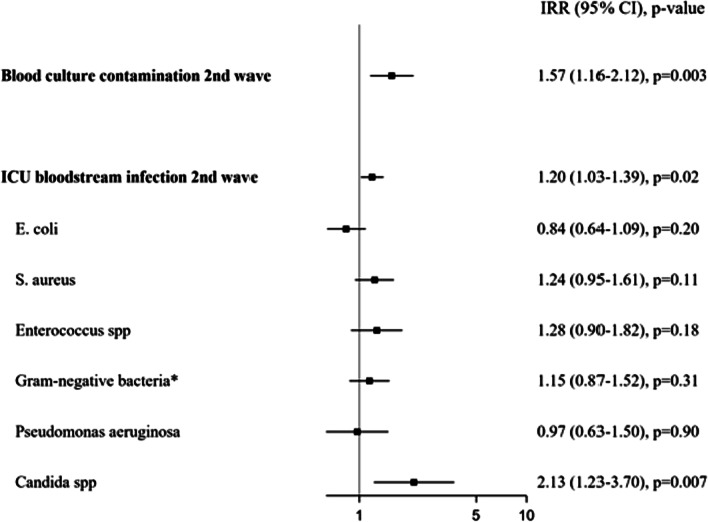
Incidence rate ratio of blood culture contamination, ICU-BSI and microorganisms during the second wave (*versus* the interim period). *IRR* incidence rate ratio, *CI* confidence interval, *ICU* intensive care unit. *E coli and *Pseudomonas aeruginosa* excluded

Using segmented negative binomial regression models, we observed an increased incidence of blood culture contaminations (IRR 1.57, 95% CI 1.16–2.12, *p* = 0.003) and ICU-BSI (IRR 1.20, 95% CI 1.03–1.39, *p* = 0.02) during the second wave compared to the interim period (Fig. 3). Moreover, an increased IRR for *Candida* spp (IRR 2.13, 95% CI 1.23–3.70, *p* = 0.007) was observed during the second wave, whereas other microorganisms responsible for ICU-BSI did not show significant trends. A confirmatory analysis on secular trends of blood culture contamination using a Poisson regression model investigating the effect of the second wave and introducing the time and the interaction between time and second wave showed similar results (Additional file 1: Fig. S5).

## Discussion

In this study, we combined two large nationwide datasets to analyze the epidemiology of positive blood culture episodes among patients in 52 Swiss ICUs over a 14-month period during the COVID-19 pandemic. After correcting for the number of patient-days, a significant increase in both blood culture contaminations and ICU-BSI was observed during the months when the ICU occupancy rates due to COVID-19 patients were the highest. Moreover, a significantly increased incidence of both blood culture contaminations and ICU-BSI was observed during the second wave compared to the interim period.

Only few studies investigated the impact of the COVID-19 pandemic on ICU-BSI epidemiology, but little is known about the role of blood culture contamination in this setting. In a before-after, monocentric retrospective study, a significant increase in the rates of contaminated blood cultures was observed among ICU patients (from 6.3 to 16.5%), mainly driven by samplings from peripheral veins [12]. In another hospital-wide study of similar design, a significantly increased rate of blood culture contamination during the COVID-19 pandemic (from 10.7 to 17.2%) was encountered; most of the episodes were, however, recorded in the emergency department [9]. In a retrospective multicenter cohort analysis in New York City, rates of positive blood cultures decreased from 3.8 to 1.6% among COVID-19 and from 8 to 5.9% among COVID-19-negative patients after exclusion of potential commensal skin microbiota [6]. Lastly, an increase in BSI due to CoNS and a decrease in BSIs due to Enterobacterales (both, central line bloodstream infection (CLABSI)-related and non-CLABSI-related) were observed in a study across five acute hospitals in London during the first wave [3].

Our results support these findings by means of a large dataset that includes 2′715 episodes of positive blood cultures collected in 52 ICUs at a national level. The vast majority of these episodes were caused by CoNS. Possible factors behind this increase include: the oversight of basic infection control practices by reallocated staff with limited or no experience in intensive care; an inadequate diligence with aseptic techniques due to ICU overcrowding; the urgency of blood sampling among critically ill patients; the implementation of ad hoc standardized protocols that envision the sampling of otherwise unneeded blood cultures; and the inexperience in wearing personal protective equipment (PPE). Contamination of blood cultures has been associated with increased antibiotic exposure, prolonged venous access, additional consultations, laboratory and diagnostic requests, prolonged hospitalizations, costs and intra-hospital mortality [17, 18]. The increased rates of contaminated blood cultures suggest, therefore, that the challenges encountered when dealing with ICU patients during the pandemic indeed negatively affected the management of critically ill patients [19]. Of note, we observed in Additional file 1: Fig. S5 an increase in blood culture contaminations at the beginning of the second wave followed by a significant decrease during the wave itself. This may be due to several reasons, included a better compliance with the hygiene measures, less overcrowded ICU, or their reorganization after the initial increase in the cases.

We found that ICU-BSI rates significantly increased during the epidemiologically more relevant second wave (*i.e.*, when the entire country was affected), although to a lesser extent than blood culture contamination. When we examined specific pathogen classes, we found a significant increase in candidemia. This may reflect the role of immunosuppressive drugs among COVID-19 patients, mainly used during the second wave, as previously suggested [20], or an increased use of central venous lines in this immunosuppressed patient population during the second wave [21].

Our study has several limitations, mainly due to its observational nature. First, we performed an ecological study using aggregated data from a microbiological surveillance system: several clinical individual patient data (e.g., baseline comorbidities, reasons for ICU-admission, source of BSI, catheter types through which blood cultures were drawn, and antibiotic treatment) were not available in both datasets. Moreover, data on COVID-19 status for each patient with a bacteremia episode were not available. Second, since the date of hospital admission was unavailable in a substantial number of episodes, the true proportion of healthcare associated infections (HAI), as well as of early and late ICU-BSI is unknown. Third, data on the number of negative blood cultures were not available, which could influence the total number of contaminations. To overcome this possible sampling bias, we used data on patient days – which may represent a surrogate of the impact of the pandemic on the ICU occupancy [22]—as denominator. Fourth, since denominator data were only partially available during the first wave, we excluded these data from quantitative analyses. However, the second wave was more prominent, hit the entire country and was therefore more representative of the national epidemiology. Fifth, due to the study design, we could not assess a potential bias due to the seasonality in the epidemiology of positive blood cultures. Finally, blood culture contamination episodes were not assessed individually but accordingly to the CDC definition, which, to the best of our knowledge, has not been validated by studies so far, thus leading to a possible misclassification bias.

These limitations notwithstanding, we believe that our analysis of nationwide data is representative and provides results that may be relevant for quality management during future pandemics. Efforts to maintain infection prevention and control measures at the highest standard even in challenging settings are paramount and could safe patient lives.

## Conclusions

An increase in blood culture contaminations and ICU-BSIs was observed during the second COVID-19 pandemic wave, especially in months when the ICU burden of COVID-19 patients was high. Our results highlight the fact that compliance with basic infection prevention and control measures should not be ignored even in challenging a challenging setting such as a pandemic.

## Supplementary Information

**Additional file 1. Figure S1**. Swiss map with the included ICU sites.** Figure S2**. Flowchart of the included blood culture episodes during the study after exclusion of episodes identified outside ICU, among children under 16 years of age or from patients abroad.** Figure S3**. Correlations of the ICU occupation due to all patients with blood culture contaminations incidence (A), ICU-BSI incidence (B) and percentage of blood culture contaminations (C).** Figure S4**. Weekly count of blood culture episodes reported to ANRESIS over the period 01.01.2018 –31.05.2021.** Figure S5**. Confirmatory analysis on secular trends of blood culture for the intermediate and second pandemic period using a Poisson regression model.

## Data Availability

The datasets used and/or analyzed during the current study are available from the corresponding author on reasonable request.

## Abbreviations

COVID-19: Coronavirus disease 2019
ANRESIS: Swiss Centre for Antibiotic Resistance
BSI: Acquired bloodstream infection
ICU: Intensive care unit
ICU-BSI: Intensive care acquired bloodstream infection
CLABSI: Central line bloodstream infection
CoNS: Coagulase negative staphylococci
CLSI: Clinical and Laboratory Standards Institute
EUCAST: European Committee on Antimicrobial Susceptibility Testing
NEQAS: National External Quality Assessment ServiceNEQAS: National External Quality Assessment Service
