# Supplementary material for: The effect of the COVID-19 pandemic on the epidemiology of positive blood cultures in Swiss intensive care units: a nationwide surveillance study
Source: Crit Care. 2021 Nov 22;25:403. doi: 10.1186/s13054-021-03814-z (PMC8607066; doi:10.1186/s13054-021-03814-z)
Supplement: Supplementary file 1 — Additional file 1. Figure S1. Swiss map with the included ICU sites. Figure S2. Flowchart of the included blood culture episodes during the study after exclusion of episodes identified outside ICU, among children under 16 years of age or from patients abroad. Figure S3. Correlations of the ICU occupation due to all patients with blood culture contaminations incidence (A), ICU-BSI incidence (B) and percentage of blood culture contaminations (C). Figure S4. Weekly count of blood culture episodes reported to ANRESIS over the period 01.01.2018 –31.05.2021. Figure S5. Confirmatory analysis on secular trends of blood culture for the intermediate and second pandemic period using a Poisson regression model. [file 13054_2021_3814_MOESM1_ESM.docx]

**Supplementary Figure 1: Swiss map with the included ICU sites**

**
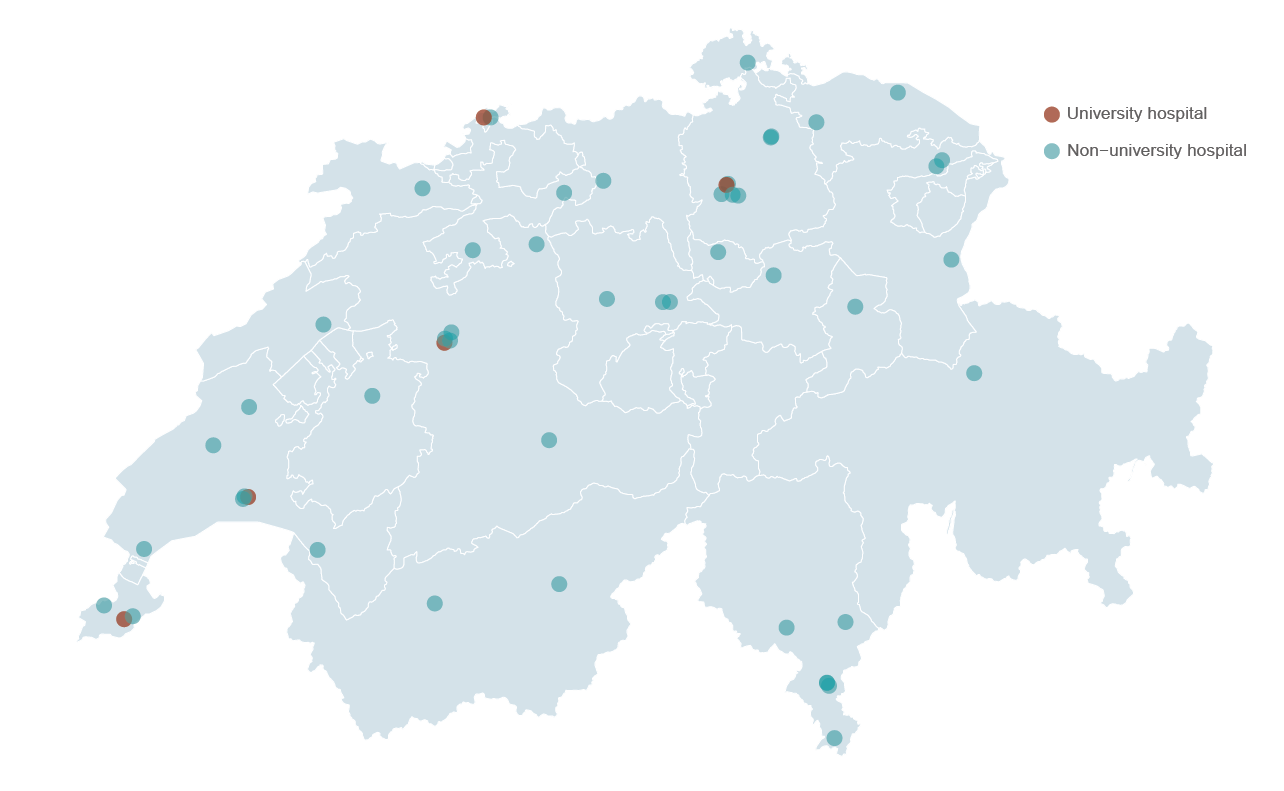
**

**Supplementary Figure 2: Flowchart of the included blood culture episodes during the study**


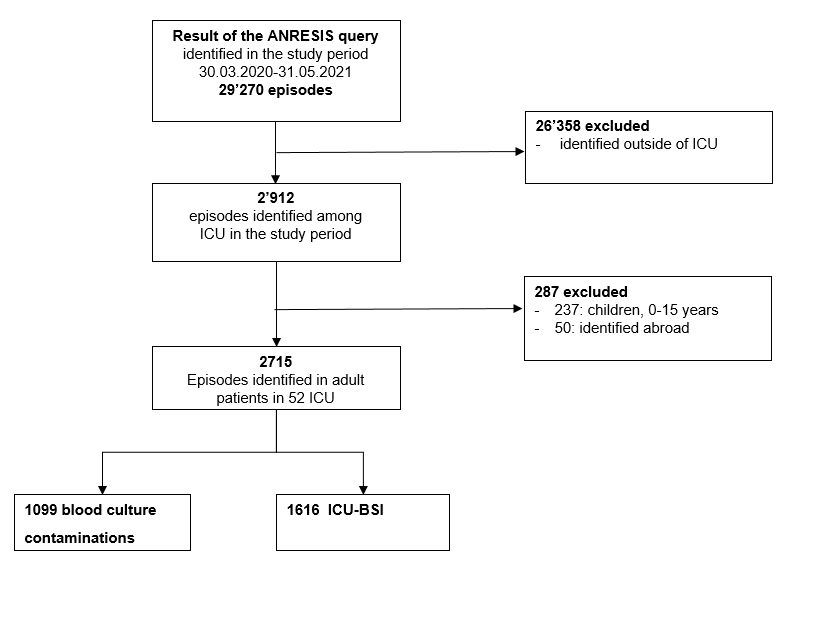
Legend. ICU: Intensive care units. Episodes identified abroad were identified in Liechtenstein.

**Supplementary Figure 3: Correlations of the ICU occupation due to all patients with blood culture contaminations incidence (A), ICU-BSI incidence (B) and percentage of blood culture contaminations (C)**


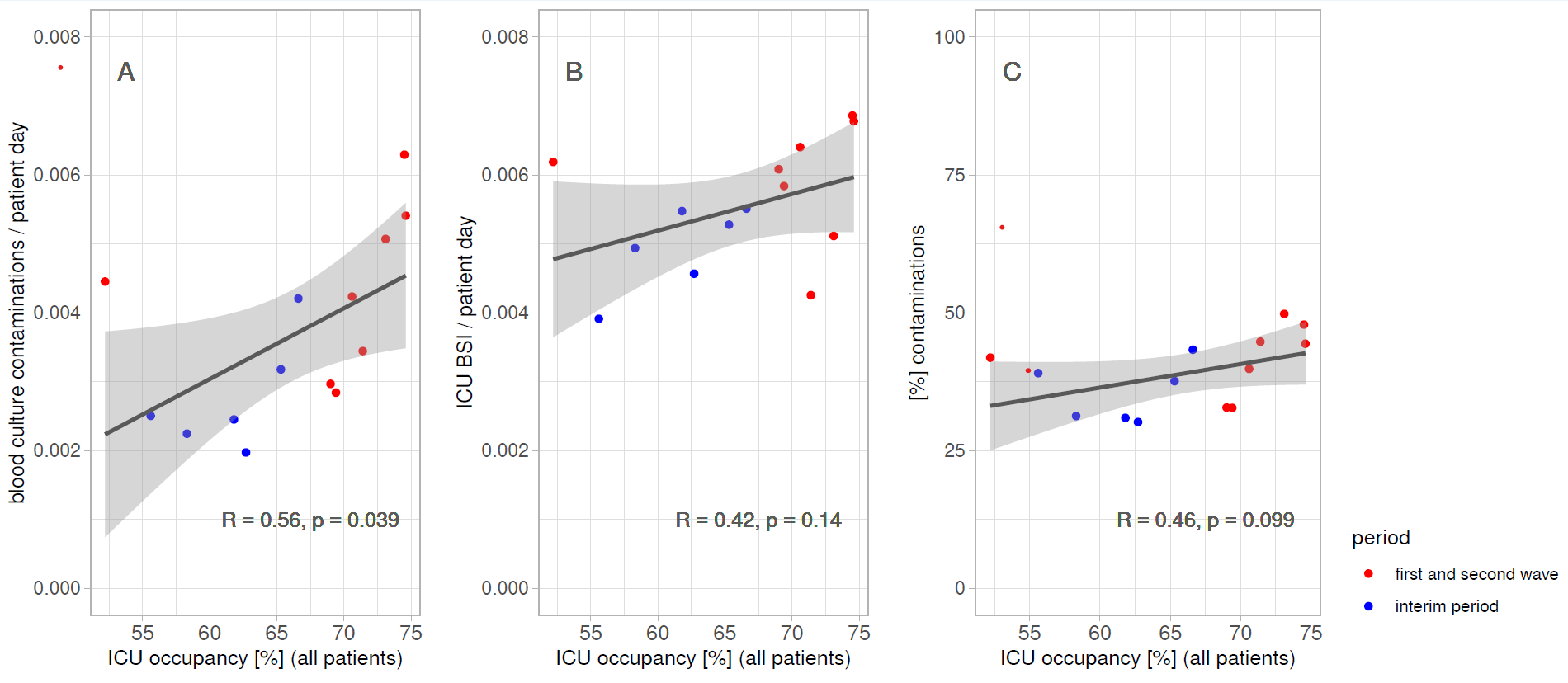


Legend: Each dot represents data from one month. In each panel a Pearson's correlation coefficient ("R"), a p-Value of a Pearson correlation test ("p"), a univariate regression line and the corresponding 95% confidence interval (gray area) are shown.

**Supplementary Figure 4: Weekly count of blood culture episodes reported to ANRESIS over the period 01.01.2018 –31.05.2021**


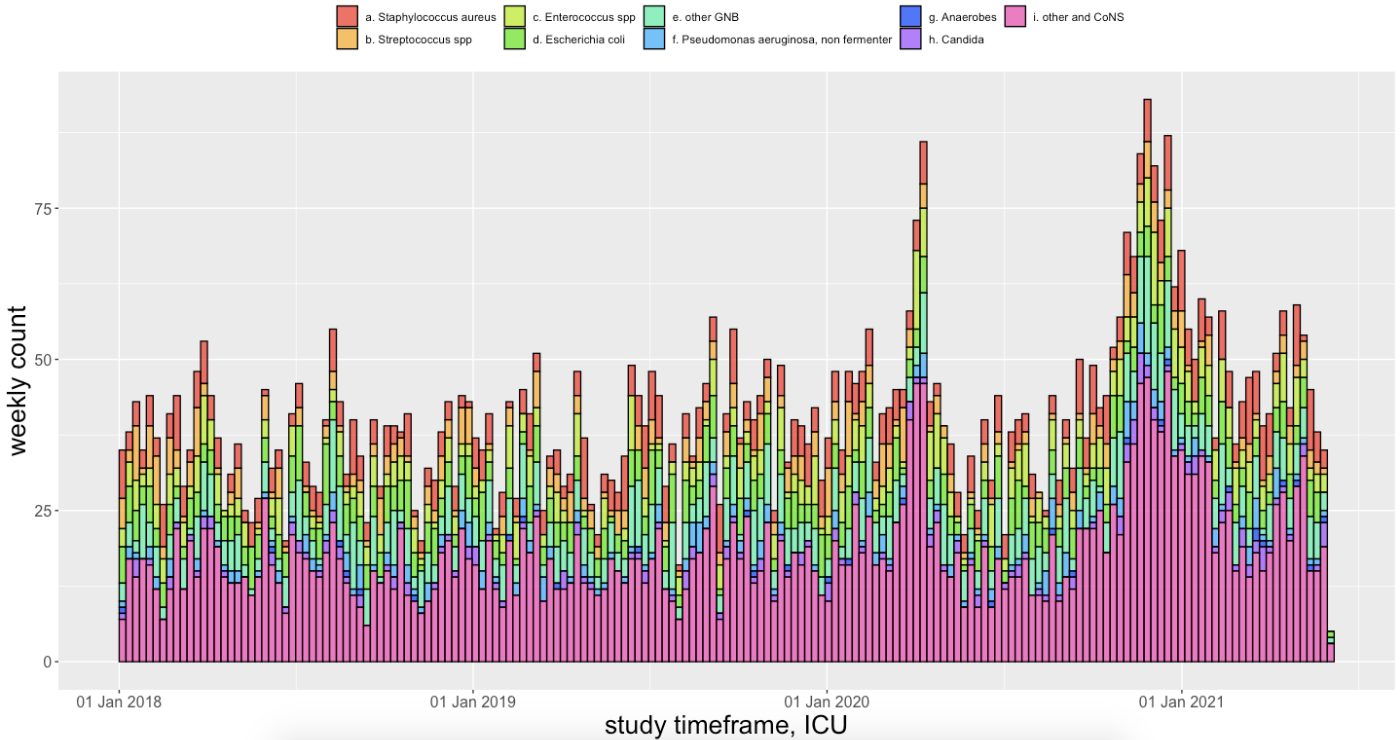


Legend. Isolates were classified in nine classes. Coagulase negative Staphylococci were classified as “other and CoNS” and represented 91% of this class (2957 out of 3263). GNB: Gram-negative microorganisms other than *E. coli* and non-fermenters

**Supplementary Figure 5: Time trends for ICU contaminant for the intermediate and second pandemic period**


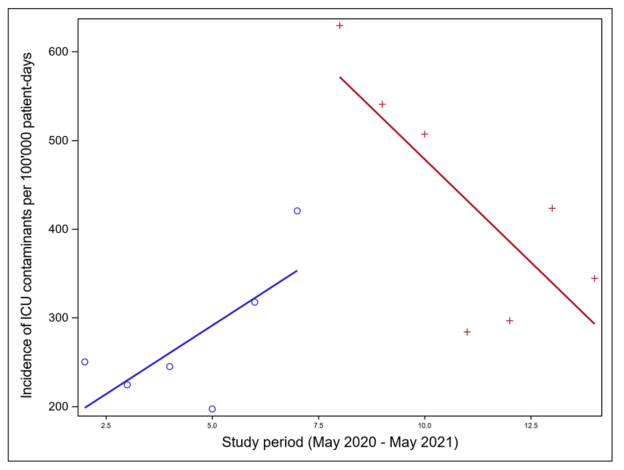


Legend. We performed Poisson regression models investigating the effect of the second wave and introducing the time and the interaction between time and second wave. In blue the intermediate period and in red the second wave period. Before the second wave (i.e., interim period), we observed an increasing linear trend of total blood culture contaminations (0.11, 95% CI 0.018-0.19, Supplementary Figure 3). The abrupt change between interim period and second wave (October-November 2020) was non-significant (IRR increase of 1.81 (95% CI 0.37-8.77). The second wave was characterized by a significant (-0.10) decreasing linear trend. The IRR after introducing an interaction between time and pandemic period was 7.90 (95% CI 3.48-17.91, p<0.001).
